# Transactions of the Illinois State Board of Health

**Published:** 1885-07

**Authors:** 


### STATE BOARD OF HEALTH.

TRANSACTIONS OF THE ILLINOIS STATE BOARD OF HEALTH.

At the regular quarterly meeting of the Illinois State Board of Health, held in the city of Chicago, April 16th and 17th, the Secretary in his usual report stated that fewer certificates entitling to practice in the State have been issued to physicians during the past quarter than during any corresponding period in the history of the Board: To graduates, upon diplomas from medical colleges in good standing, 116, and to 2 others upon examination in branches omitted by their respective colleges; also 2 to non-graduates upon proof of over seventeen years' practice in the State. There were 22 applications for certificates rejected through failure to comply with the requirements of the Board—which is also less than the usual proportion of such cases.

MEDICAL EDUCATION.

As it is during this quarter that the new graduates in medicine begin to appear, their applications have been watched with more than usual interest at this time, as affording some clue to the effect of the new schedule of requirements.

Although 78 medical colleges out of the 116 in the United States, claim to have exacted, at their last session, a preliminary-education requirement as a condition of matriculation, the evidence afforded by the applications thus far received goes to show that in too many cases the standard of such education must be very low.

From the graduates of one college, which announces that a preliminary examination will be held in accordance with the rules of the State Boards, five out of the seven applications received contain such intrinsic proof of defective preliminary education, that they were presented to the Board.

[The following is a verbatim copy of a typical specimen of the applications submitted:

I send to you my Diploma for your approval the degrees Doctor of Medicine was confered upon me at the ____________________ annual Commencement of the ________________________ held on Teusday Mar the ____ 1885. you will pleas return as soon as possiabl and oblige. Enclosed you will find the fee for Regestration.

Yours truly

P S if the fee is not suficient I will forward the balance if you notify me by card I understood the fee was one Dollar.

I presume the enclosed Papers will be all that will be requierd. return Diploma to Me at ____________________ Illinois ________________ co.

In another case the first letter, dated March 9, contained a request for a blank affidavit, which was, of course, immediately forwarded. The signature to the affidavit, received March 14, was so evidently not by the writer of the first letter—which was unexceptionable in every respect—that some delay occurred, pending inquiry. Under date of March 30, the applicant, in a handwriting differing both from that of the letter of March 9 and from the signature to the affidavit, writes:

I send you my Deploma today by Expr. and letters of recommendation. I sent fee and affidavit Bout three weeks since. I would like to know at once whether or not to expect a Certificate. you will please return Deploma by Exp.

It is possible that this young gentleman—who, by the way, states that his school of practice is "Alapathy"—passed his entrance-examination as he conducts his correspondence—that is, by proxy.]

There is other evidence—some in the form of direct charges by rival schools, some by the students themselves—that the entrance-examination is, in many cases, a mere form. Colleges which, on inquiry, claim to exact proof of general preliminary fitness and capacity for the study of medicine, evade the spirit and intent of the requirement in a variety of ways. In some instances it is announced that the examination is confined to applicants who intend to practice in the States in which such examination is made a test of the good standing of a college. In other cases the requirement is entirely omitted from the college announcement, but graduates intending to practice in the States just described are furnished special certificates, setting forth that they have undergone the preliminary examination demanded. Still another form of evasion consists in announcing that a preliminary examination is exacted, but coupling this announcement with a long list of equivalents which will be accepted in lieu of such examination, beginning with the diploma of graduation from a good literary and scientific college or high school, or a first-grade teacher's certificate (the only substitutes which the Board accepts), and ending with a "special arrangement" whereby the certificate of a preceptor is accepted as sufficient evidence.

It is not to be inferred, however, that there has been no gain or improvement in the standard and methods of medical education since this schedule of minimun requirements was first published. On the contrary, probably all has been accomplished that could have been reasonably expected. But these illustrations will indicate the scope and methods of the antagonism aroused by a measure for improvement, the first effect of which results in diminished classes and loss of income—an antagonism natural enough, perhaps, in view of the largely commercial character of many institutions of medical education. They also serve to show the necessity for further earnest and continuous effort.

In this direction it is suggested that one of the tests of the good standing of a medical college, for the purposes of the Medical-Practice Act in this State, should be its honest and unequivocal statement, in annual announcement and elsewhere, of the requirements for admission to its lecture-classes, and an honorable and impartial enforcement of such requirements.

To secure the recognition of its diplomas in this State—as in good standing—every college should distinctly announce that the conditions of admission to its classes are: 1. Credible certificates of good moral character. 2. Evidence of the possession of a good English education, including mathematics, English composition and elementary physics, or natural philosophy—such evidence to be furnished, in the absence of a diploma or certificate of graduation from a good literary and scientific college or high school or a first-grade teacher's certificate, by a thorough examination in the branches of such education. While this is the minimum, as to preliminary education, which should be distinctly specified and impartially exacted, it does not preclude colleges, from making other requirements as conditions of admission to their lecture classes.

The Secretary also suggests that the affidavit now required by law as to the lawful possession of a diploma, may be properly modified so as to include the information necessary to aid the Board in deciding whether such diploma was issued by a legally-chartered medical institution in good standing, as the third section of the Medical Practice-Act requires it to be—such modification to consist, substantially, in striking out the words "said institution," with which the declaration ends in the affidavit now in use, and substituting therefor the words *a legally-chartered medical institution in good standing, as defined by the Illinois State Board of Health, in its Schedule of Requirements printed on the back hereof.* A foot-note on the face of the affidavit should call the especial attention of the affiant to the import of this declaration.

The law specifically requires that applicants for certificates shall furnish this proof—proof not merely that their diplomas are from legally-chartered medical institutions, but that they are from such institutions " in good standing." Obviously the graduate of a given institution is a competent, if not the best, witness as to the qualifications, exacted of him before he received its diploma. And since these qualifications go to the essence of the question as to the standing of the institution, it is submitted that the Board should avail itself of this form of proof.

Acting upon these suggestions the Board subsequently during the meeting adopted the following resolution:

CONCERNING MEDICAL EDUCATION.

WHEREAS, Many medical colleges do publicly announce that an entrance examination of candidates for admission to their lecture courses will be exacted, and do honestly and impartially enforce such examination; while, on the other hand, a number of schools either avoid making such announcement, or evade the practical enforcement of any requirement of general education preliminary to the study of medicine; and

WHEREAS, These conflicting practices result in lowering the standard of medical education by attracting to a certain class of schools students who are poorly prepared for the study of medicine; therefore be it

*Resolved*, That, in order to secure the recognition of its diplomas as in good standing for the purposes of the Medical Practice Act in this State, it is necessary that each college shall distinctly state in its annual announcement that the conditions of admission to its classes are: 1. Credible certificates of good moral character. 2. Diploma of graduation from a good literary and scientific college or high school, or a first-grade teacher's certificate. Or, lacking this, a thorough examination in the branches of a good English education, including mathematics, English composition, and elementary physics or natural philosophy.

*Resolved*, That the Secretary of the Board be, and hereby is, instructed to furnish a copy, of the foregoing preamble and resolutions to the Dean or Secretary of every medical college, and to the editors of medical journals, in the United States.

On motion of Dr. Mackenzie it was also

*Resolved*, That, since the publication of the names and addresses of matriculates is desirable for purposes of information, the Secretary be authorized to request of all colleges desirous of being accounted in good standing in this State, that they publish in their successive annual announcements complete lists of the matriculates, as well as of the graduates, of each immediately preceding session.

THE CHOLERA.

In connection with the efforts made to secure information from the National authorities concerning the status of cholera abroad, attention is called in the report in the cable dispatches received by the newspapers during the meeting announcing that the French, Italian and Portuguese governments had ordered a quarantine of detention against Spanish vessels; and the appearance of the disease at Jaen, in the province of that name, in the south of Spain, and at Santiago de Compostela, in the extreme northeastern province of Coruna—the same dispatch saying that the panic in Spain over the spread of cholera is increasing as reports continue to arrive showing that new points are being constantly attacked; that the government is taking energetic measures to isolate infected towns; and that a circular of warning has been sent by telegraph to the authorities of all the provinces cautioning them against the admission of persons or goods from twelve specified towns, all of which are officially stated to be more or less infected.

Simultaneously with this latter information the first official statement was made public by the Secretary of State, who announced on the 18th of April, the receipt of a dispatch from the United States consul-general at Madrid, saying "that he is informed by the director-general of health that there is no cholera in Spain, and that the cases recently reported in the province of Valencia are not cholera." The Spanish government has instructed its embassidors to protest against quarantine restrictions, and a dispatch of the 19th inst., from Barcelona also asserts that the disease is not Asiatic Cholera, but cholera morbus or cholerine due to local causes, the outbreak at Alcira, near Valencia, for example, being caused, it is claimed, by the failure of the regular water supply, in consequence of which "the people have been drinking from a canal which was tainted by paper mills that use suspicious rags."

In view of these contradictory statements, and in the absence of full and authentic information from the National health authorities, sanitarians are justified in regarding, for precautionary purposes, the disease now so widely spead through the littoral provinces of Spain as true Asiatic Cholera, and in apprehending present danger of its introduction into this country through commercial intercourse with the Spanish possessions in the West Indies—Cuba, Porto Rico, etc.—and less directly with those in South America.

Attention is also called to the fact that the country is threatened with an influx, by imigration from Italy, of a people reduced to the verge of beggary and starvation by last year's cholera epidemic and its results. The low rates of passage will tempt to violation of the law against overcrowding, with all the suffering and unsanitary conditions which will thence result. The poverty of the people and their modes and habits of life will add to the evil; and increased burdens and responsibilities will be thrown upon the authorities of every port at which these immigrants land, as well as upon the communities in which they may settle. These considerations may make it necessary to begin the work of sanitary supervision of travel and quarantine along the State boundary lines earlier than would otherwise be necessary. Already the first instalment of the Italian immigration has arrived in Chicago.

The Board adopted the following preamble and resolutions concerning these matters:

CONCERNING INFORMATION OF FOREIGN EPIDEMIC DISEASES.

Whereas, Prompt, full and trustworthy information of the existence of epidemic diseases, such as Asiatic cholera, yellow fever and small-pox, in the foreign ports in commercial relations with this country, is a matter of the first importance to the success of efforts for preventing their introduction or limiting their spread; and

WHEREAS, It is understood that, under the authority conferred upon the President by Sec. 1752 of the Revised Statutes of the United States, consular officers and other foreign agents of the General Government are required to furnish such information : Therefore, be it

*Resolved*, That the Secretary of this Board be, and he hereby is, instructed to respectfully request of the honorable the Secretary of State that he cause to be transmitted to the office of this Board, at Springfield, so much of such information as may be useful in guiding action for the protection of the people of this Commonwealth against Asiatic cholera, yellow fever and small-pox.

On motion of Dr. Clark it was also

*Resolved*, That the State Board of Health of the State of Illinois respectfully but earnestly requests the President of the United States to authorize the National Board of Health to use so much of the contingent epidemic fund, appropriated by the last Congress, as may be necessary for preparing and enforcing an adequate system of preventive measures against the introduction and spread of foreign pestilential diseases in co-operation with, and in aid of, State and local health organizations and with especial reference to Asiatic cholera.

*Resolved*, That the Secretary be authorized to transmit a copy of this resolution to the President.

STATE SANITARY SURVEY.

As already stated, the first distribution of the blank inspection returns and accompanying instructions, embracing an aggregate of about 270,000 houses, was completed during the last week of the quarter. This distribution began with Alexander county and progressed northward to the tier of counties along the Wisconsin line, which was reached in ample time to prepare for work as soon as the weather would permit. Before the middle of the State was reached, responses began to be received from localities in the southern counties, and by the close of the quarter 143 towns had been heard from. General publicity has been given to this effort by the press of the State, to secure which special circulars were addressed to the editors of 775 different publications.

A blank form for a tabular statement of these inspections has been prepared and printed, and is now ready for distribution. These will be furnished in duplicate sets, one to be returned to the office of the Board. They will show, at a glance, the actual sanitary condition of any given house and premises at the date of inspection; and, in the event of Asiatic cholera, or other epidemic contagious disease, making its appearance in a locality, they cannot fail to be of great practical value, not alone to the authorities of such locality, but also to the Board, in indicating, without loss of time, the direction and manner in which its coöperation, advice or authority may be best employed.

Circular-letters have been addressed to the managers of all railroad companies operating in this State, urging immediate attention to the condition of the water-supply and of privies and water-closets at stations, shops, roundhouses and other places along their respective roads. Satisfactory replies have been received from nearly every company addressed, and some of the most important have already practically completed the required work.

County boards and officers in charge of public institutions—alms-houses, jails, asylums, etc.—have also been requested to secure a thorough sanitary policing of such places, and specific instructions on the most important points have been furnished.

Copies of the blanks, forms, etc., prepared for this general work, have been furnished, on request, to the health department of Chicago; and an excellent form of inspection return, modified to meet the city conditions, has been prepared by the Commissioner of Health, Dr. DeWolf, for an inspection of some 70,000 houses. It is proposed to complete the inspection of 50,000 of these, and to secure the correction of the defects thus disclosed, by the 1st of June.

One of the encouraging indications of the interest taken in this effort to prepare the State against an epidemic is found in the large number of letters from private individuals asking advice, making suggestions, or expressing approval.

SANITARY CONDITION OF CHICAGO.

During the Friday morning session, Dr. O. C. DeWolf, Health Commissioner of Chicago, was present by invitation to speak upon the sanitary condition of the city, the work in progress and projected, and the preparations for cholera. From the standpoint of the sanitarian, Dr. DeWolf said, Chicago was a clean city, although its muddy streets made it seem dirty. Its low death-rate and the failure of small-pox to spread, notwithstanding 35 introductions of the contagion since last June, showed it to be clean in a sanitary sense; and the work now in progress and projected would, he believed, make it clean in appearance as well as in fact. Referring to the house-to-house inspection in the State at large, he said that the health department was also inspecting at the present time about a thousand houses a week in the worst quarters of the city; in a short time this would be increased, so that by the middle of June he hoped to have all that really required supervision thoroughly inspected and put in good condition. Some 9,000 tenement houses, which are usually a serious sanitary evil in all large cities, are under constant supervision, and he believed them to be as unobjectionable as it was practicable to make such buildings. If cholera should come, the preparations were already completed to promptly take charge of the first cases, to furnish medical attendance and nurses, to depopulate an infected house or locality, and to carry out whatever measures were necessary to prevent any spread.

The thanks of the Board were tendered Dr. DeWolf by the President, for his very interesting and reassuring statement.

At the afternoon session Mr. O. C. Guthrie, of Chicago, presented, by invitation, a brief outline of his plans for the sewerage and drainage of Chicago and its suburbs. Their important features embrace a study of the hydraulics of the

Desplaines river, with reference to the effect of high water upon the cleansing of the Chicago river through the canal (by counteracting the action of the pumps), upon the integrity of the canal itself, and upon the safety of Chicago and Joliet from inundation.

DISINFECTION AND DISINFECTANTS.

The Secretary asked leave to submit a copy of the Preliminary Report on Disinfection and Disinfectants made by the Committee on that subject appointed by the American Public Health Association, stating that, as Secretary of the Sanitary Council of the Mississippi Valley and chairman of its special committee on General Sanitation, he had, in accordance with a resolution of the council, addressed a letter, March 14th, to Surgeon George M. Sternberg, U. S. A., chairman of the Committee on Disinfectants, requesting to be furnished with "a plain, practical paper on disinfection and disinfectants for popular use and distribution." Surgeon Sternberg, under date of April 14th, in a letter accompanying this copy, writes: "At a special meeting of the Committee on Disinfectants, called for the purpose, the paper submitted was carefully considered and adopted unanimously as expressing the views of this committee with reference to the best methods of disinfection known to us."

As this paper was intended for popular use and distribution the Secretary remarked that he knew of no better way of securing that end than by publishing it in the report of the proceedings of the Board.

Dr. Ludlam moved that the copy of the Preliminary Report be received and published as suggested. Carried. It was subsequently ordered that the Secretary prepare a special edition of the paper, embracing only the practical instructions, for the use of local boards of health and health officers throughout the State.

During the sessions the following action was taken upon cases arising under the

### MEDICAL-PRACTICE ACT.

Dr. Charles McLean, of No. 182 West Madison street, corner of Halsted, Chicago, charaged with unprofessional and dishonorable conduct in (1) Fraudulently issuing a card in the following terms :

*Dr. McLean's Medical Institute for Female Diseases, 182 West Madison street, Room 11. Phrenology and Mesmerism Successfully Taught. Terms reasonable. President—Charles McLean, M. D., assisted by Prof. L. H. Anderson and others. Dr. McLean's New Developer for Delicate Ladies never fails to Strengthen the Chest, Develop the Bust, and Beautify the Complexion.*

(2) Attempting to blackmail a female patient, attracted by the foregoing card, and whose testimony before a Chicago police justice, submitted to the Board, established the fact of grossly indecent solicitation and practice on the part of said McLean. (3) Obtaining money under false pretencees, for which he was arrested, confined in jail, and released on refunding the money. (4) Grossly immoral conduct and general reputation in the building where his so-called "Institute" was located.

In a pamphlet styled "The Mind Cure," dated October, 1884, appeared the following :

"We duly appreciate an interesting call and visit by Charles McLean, M. D., LL. D. The doctor is widely known in Europe and America. Is a graduate of Glagow University, Scotland, in the O. S. ; also a graduate of one of the Chicago medical colleges, N. S. Has been a student of law and theology, and in the latter received D. D., but chooses not to use it, having retired from this field after two journeys around the world. He is an author of some repute, and speaks several languages. He is now a resident of Chicago. Full of energy, has gained wealth and prominent standing among druggists here and across the water. We find him cultured and progressive. He had learned of our new association and purpose and spoke so highly of the Mind Cure system we told him our door was open if he could identify with it. He is now a member of our association and a contributor to its finances. Also to be an editorial contributor to our columns. Such steps are portentous. Let them come. The doctor now is president of a Medical Institute at 182 West Madison street and will be heard from."

Dr. McLean had been duly notified to appear in answer to the foregoing charges, but it was stated by one of the sworn witnesses that he had disappeared after being released from jail. After a full consideration of the evidence presented, it was ordered that certificate No. 5853, issued to Dr. Charles McLean on the 13th of March, 1883, be revoked.

Dr. James F. Cook, of the "St. Jacob Institute," No. 14 South Clark street, Chicago, charged with unprofessional and dishonorable conduct in (1) Being associated with a fraudulent medical "institute," so-called, of which he advertises himself to be the "superintendent. (2) Falsely testifying as to the physical condition of one Smith Whittier, for the purpose of mitigating the sentence of said Whittier, convicted in the United States District Court of sending obscene matter through the mails. (3) Writing, publishing and distributing an obscene pamphlet entitled "Hidden Secrets."

Dr. Cook had been duly notified to appear in answer to the foregoing charges, and after full consideration of the evidence submitted, it was ordered that Certificate No. 527, issued to Dr. James F. Cook on the 2nd of January, 1878, be revoked.

Dr. J. Alonzo Greene, formerly of St. Louis, Mo., now at 34 Temple Place, Boston, Mass., charged with unprofessional and dishonorable conduct in (1) Falsely and fradulently claiming to have established "a system of *curing all forms of chronic or lingering diseases*." (2) Falsely and fraudulently advertising that the Illinois State Board of Health, among other Boards, Colleges and Medical Societies, had awarded him and his associates "diplomas for their remarkable skill in curing disease, and their discoveries of valuable remedies." The evidence being deemed conclusive as to the truth of the charges, it was, after due consideration,

Ordered, That Certificate No. 4811, issued to Dr. J. Alonzo Greene on the 17th of December, 1880, be revoked.

Dr. Joseph Atherton, of East Paw Paw, DeKalb county, whose certificate was revoked October 5, 1882, and who had asked an opportunity to present reasons for its restoration, stated that he acknowledged the sufficiency of the grounds upon which his certificate had been revoked, but claimed that he had been misled, and upon discovering his mistake and its consequences had abandoned the objectionable practices. He asked the Board to restore his certificate, pledging himself to act in conformity with the requirements of the Medical-Practice Act as to honorable professional conduct, and sustained his application by strong commendatory letters from physicians and others. On motion of the Secretary, Dr. Atherton's certificate was ordered to be restored.

On motion of Dr. Mackenzie, all other cases under the Medical-Practice Act, not otherwise disposed of, were continued to a special meeting to be called by the Secretary at an early date.

At 9 o'clock P. M., Friday, the annual examination of candidates was concluded. The Secretary announced that two of the class of nine had withdrawn without attempting to pass the preliminary examination upon general education. Of the remainder, the following gentlemen attained the required percentage entitling them to pass:

ADOLPH C. BRENDECKE, Chicago.
JONATHAN E. CLARK, Mason, Effingham county.
JAMES D. CRAIG, M. D., Rogers' Park, Cook eounty.

The following is the schedule of questions submitted at this examination, and to which eighty per cent. of correct answers was required in order to pass.

### ILLINOIS STATE BOARD OF HEALTH.

*Quarterly Meeting, Chicago, April 16-17, 1885.*

---

### EXAMINATION IN ANATOMY.

BY W. HASKELL, M. D.

1. Describe the frontal, sphenoidal and maxillary sinuses. Which ones exist in infancy?
2. Give the anatomy of the shoulder joint.
3. Describe a rib.
4. Describe the muscles concerned in pronation and supination of the fore-arm.
5. Name the branches of the external carotid artery.
6. Describe the circulation of the liver.
7. Describe the brachial plexus, giving the nerves of which it is formed, its location and its branches.
8. Name the arteries, veins and nerves cut in an amputation of the leg in its middle, and give their relations.
9. Give the anatomy of the eye.
10. Give the anatomy of the prostate gland.

### EXAMINATION IN PHYSIOLOGY.

BY W. R. MACKENZIE, M. D.

1. Describe the physical properties of the blood.
2. Its chemical composition.
3. Its structural character.
4. Its pulmonic and its systemic circulations.
5. Its circulation in the fœtal heart.
6. Describe the function of respiration.
7. What are the sources of carbonic acid in the body ?
8. State the normal relation of the respiratory act to the heart-beat.
9. Describe digestion.
10. What is the function of the liver in the process of nutrition ?
11. Give the composition and properties of chyle.
12. What is bile—its source and function ?
13. What is secretion, and name the more important secretions of the body.
14. What is the specific gravity of normal urine ?—its reaction.
15. What is the normal temperature of the human body, and what the minimum and maximum temperatures consistent with life ?
16. What are the general functions of the great sympathetic or tri splachnic nerve ?
17. What is sensation, and what are its various kinds or modes ?
18. Mention the nerves concerned in the special sense of taste, and their respective offices.
19. Describe the physiology of sleep.
20. What is meant by somatic death, and to what is it due ?

### EXAMINATION IN CHEMISTRY.

BY A. L. CLARK, M. D.

1. What are the general properties of acids ?
2. What is a salt?
3. Give the chemical symbols for tin, lead, potassium, sodium, iron, gold, silver, sulphur.
4. What is catalysis ?—synthesis ?
5. What is a quantitative analysis ?
6. In what way would you test water for organic impurities ?
7. To what chemical element in nitro-glycerine is due the explosive nature of that substance ?
8. How are the gases forming air united ?
9. Give the general properties of chlorine.
10. What is tetrad ?
11. What is meant by the diffusion of gases ?
12. Will the air at the top or bottom of a room contain the more carbonic acid gas, and why ?
13. What fluid is the most universal solvent ?
14. What is meant by the terms osmosis and exosmosis ?
15. What are incompatibles ? Give one or more illustrations.

### EXAMINATION IN PATHOLOGY.

BY R. LUDLAM, M. D.

1. Define pathology as distinguished from physiology.
2. What is the difference between fever and inflammation ?
3. Give the chief and common characteristics of the malignant diseases.
4. Define the difference between a general and a local disease, and give illustrations of each.
5. Explain the abdominal tympanitis of typhoid fever.
6. Describe the pathology of apthous ulcération.
7. What is emphysema, and what is its most frequent cause ?
8. Give the pathological location of inflammation.
9. Which lung is most frequently inflamed, and why ?
10. Why does pelvic congestion frequently alternate with portal congestion ?

### EXAMINATION IN MATERIA MEDICA AND THERAPEUTICS.

BY GEO. N. KREIDER, M. D.

1. Give the habitat, preparations, and therapeutic uses of erythroxylon coca.
2. What salt derived from it has been recently found valuable in therapeutics ? Give its uses.
3. Describe the methods of producing two kinds of electricity.
4. Define the therapeutic indications of each.
5. Give the therapeutic uses and applications of cold.
6. Of heat.
7. What is your treatment of chronic constipation, and advice ? Give your prescriptions.
8. Write a prescription for the first stage of cholera.
9. Give the composition and doses of five officinal preparations of opium.
10. How would you administer quinine other than by the mouth? Give two ways.
11. What general anæsthetic do you prefer ? Describe your method of using it to produce anæsthesia, giving the precautions you would take.
12. Give the adult doses of cod-liver oil; chloral; oil of turpentine; asafœtida; salicylic acid; tincture of aconite; tincture of digitalis; solution of arsenite of potassium.
13. Give the officinal name and composition of Dover's powder; Fowler's solution; mercury and chalk; seidlitz powder; blue ointment; compound rhubarb powder.
14. What doses of compound tincture of opium would you give to children one, three and four years old, respectively ? How much opium would each receive ? How do you compute the dosage for children ?
15. For poisoning by what drug would you give freshly prepared hydrated sesquioxide of iron ? How you prepare it ?

### EXAMINATION IN HYGIENE.

BY JOHN H. RAUCH, M. D.

1. Name the principal preventable diseases in the order of their greatest fatality.

2. Give the differential diagnosis of small-pox—of scarlet fever—of measles—of chicken-pox.

3. What diseases are spread mainly through the medium of water—what mainly through the air—and what mainly by direct contact ?

4. At what period of life is the death-rate the highest, and what are the principal preventable causes of such mortality ?

5. Describe the operation of vaccination—its phenomena—complications—the relative merits and demerits of bovine and humanized virus—and state the ages at, or conditions under, which the operation should be repeated.

6. What precautionary measures would you, if a medical officer of health, enforce in a community threatened with Asiatic cholera ?

7. What measures would you advise on the appearance of a first case of Asiatic cholera in your community ?

8. What are the most common sources of pollution of a water-supply in large cities, and what in small towns, villages, and in the country ?

9. What is the distinguishing characteristic of sewer-contaminated water ?

10. What effect does boiling have upon a contaminated or impure water ?

11. Mention the desirable sanitary conditions of a site for a dwelling—character of soil—drainage—exposure—elevation.

12. What are the pathological indications of defective sewerage and drainage of a house and premises ?

13. What are the physical indications of the same condition ?

14. What is ground air, and how may it be deleterious to health ?

15. Within what distance of a well, or other source of water-supply, is a privy-vault dangerous in a sandy soil—in a loam—in clay ?

16. What effect would general subsoil drainage produce upon the temperature, atmospheric humidity and wind movement of this State ?

17. Formulate, briefly, a set of rules for school hygiene.

18. Mention four of the best disinfectants—state their applications—and give directions for their use.

19. Define the difference between an antiseptic and a germicide.

20. What diseases of animals are communicable to the human family ?

### EXAMINATION IN MEDICAL JURISPRUDENCE.

BY JOHN H. RAUCH, M. D.

1. Define medical jurisprudence, and state what degree of knowledge the law expects of a medical witness ?

2. What is the duty of a medical witness as to questions of fact ?

3. What is his privilege as to questions of expert knowledge opinion ?

4. What are the important points to observe, with reference to the subject, in conducting a post mortem for legal purposes?

5. Describe the signs which would enable you to determine whether an incised wound had been inflicted before or after death?

6. What portions of the body would you select for a chemical examination in a case of suspected poisoning?

7. What are the indications of viability in the fœtus, and at what age are these found?

8. In suspected infanticide, what proof would determine respiration?

9. Describe the course of procedure in commitment to an insane asylum in this State.

10. What important legal bearing has the collection of vital statistics?

